# Factors associated with syphilis screening uptake among pregnant women in health facilities in Brong Ahafo Region of Ghana

**DOI:** 10.1186/s40748-015-0009-2

**Published:** 2015-03-18

**Authors:** Damien Punguyire, Emmanuel Mahama, Timothy Letsa, Patricia Akweongo, Bismark Sarfo

**Affiliations:** Department of Epidemiology and Disease Control, Municipal Health Directorate, Techiman, University of Ghana, Legon, Ghana; Department of Epidemiology and Disease, Kintampo Health Research Center, University of Ghana, Legon, Ghana; Department of Epidemiology and Disease, Regional Health Directorate, Brong Ahafo Region, University of Ghana, Legon, Ghana; Department of Epidemiology and Disease, School of Public Health, College of Health Sciences, University of Ghana, Legon, Ghana

**Keywords:** Antenatal care, Syphilis screening, Transmission, Congenital syphilis, Prematurity

## Abstract

**Background:**

Congenital syphilis is a consequence of undiagnosed, untreated, or inadequately treated maternal syphilis and results in serious adverse outcomes. It is easily diagnosed and in Ghana it is treated at points of care free; yet most pregnant women attending antenatal clinic (ANC) in Ghana are not screened. This study identified some factors influencing syphilis screening uptake at medical facilities during pregnancy in the Brong Ahafo Region of Ghana.

**Method:**

A cross-sectional study was conducted in two districts in Brong Ahafo Region of Ghana. All the health facilities in the selected districts that run antenatal services were assessed on their preparedness to screen syphilis for pregnant women. Interviews were conducted among 390 pregnant women attending ANC at five hospitals in the two districts to identify individual and community level barriers to syphilis screening.

**Results:**

In all 37 health facilities conducted antennal clinics in the two districts in 2013, 75.7% of the health facilities were public; Techiman had the higher number of health facilities (64.9%), test kits were available in only 29.7% of the health facilities and 43.2% of 37 health facilities were conducting syphilis screening. Majority of the pregnant women (57.7%) were within the age range of 20–29 years, 53.1% were in their third trimester, 32.6% in second trimester and 14.3% were in the first trimester. Syphilis screening was 52.4% among 37 health facilities. Among 390 pregnant women who participated in the study syphilis screening was 21.1%. At the health facility level, screening was significantly associated with the type of hospital (whether private or public), availability of test kits, and trained personnel, such as doctors/midwives and syphilis education. At the individual level, attending a public hospital (OR=5.49; 95% CI=1.71-17.65), willingness to request screening (OR=2.72; 95% CI=1.09-5.88), and being in the third trimester of pregnancy (OR=16.47; 95% CI=2.02-132.81) were significantly associated with syphilis screening uptake.

**Conclusion:**

Despite government’s free screening policy for syphilis among pregnant women, the coverage of antenatal screening is still low. Training of lower level health workers and regular supply of logistics are crucial for the success of the syphilis prevention programme.

## Background

Congenital syphilis is considered a disease of major public health importance and it is associated with stillbirths, perinatal deaths, prematurity and congenital infections [1,2]. Close to 70% of active syphilis among pregnant women is associated with adverse pregnancy outcomes [3]. Among women attending antenatal clinics in Africa, estimates of syphilis sero-reactivity range between 4 and 15% with about 494,000 infants dying of congenital syphilis each year [4].

Onsite screening for syphilis at antenatal clinics with immediate treatment for infected women could dramatically improve treatment rates and reduce the burden of the disease for both mothers and fetuses. As part of the global campaign towards elimination of congenital syphilis the World Health Organization (WHO) recommends an increase in antenatal base screening and treatment of pregnant women and their sexual partners for syphilis to 90% by 2015 [5]. Unfortunately less than 40% of pregnant women attending antenatal clinics across sub-Saharan Africa are being tested for syphilis [6].

In Ghana, the national AIDS/Sexually transmitted infection Control Program (NACP) was established as part of the national response to the acquired immune deficiency virus (HIV) pandemic with oversight responsibility for managing the prevention and treatment of sexually transmitted infections (STIs). Annually, syphilis test kits are provided free of charge to all health facilities providing prevention of mother-to-child transmission of HIV/AIDS (PMTCT) services to ensure all pregnant women are tested at the same time that they receive HIV test so as to eliminate the barriers to screening when conducted in the laboratory. In spite of that, less numbers of women are tested for syphilis than HIV. For example, in 2011, only 40% of all antenatal clinic (ANC) registrants across the entire country were screened for syphilis [NACP 2012 Annual report, unpublished observation]. Incidentally, Brong Ahafo region recorded just about 41% of syphilis screening in the same year. This coverage is far lower than expected where ANC attendance is usually 90% or higher. Meanwhile, a study conducted in the Ashanti region among 210 antenatal clinics showed only 3.3% of facilities routinely screened pregnant women for syphilis [7]. The objective of this study is to identify factors influencing low syphilis screening in the Brong Ahafo region of Ghana which may help improve screening uptake at the ANC level and how this can be sustained over time to reduce the burden of syphilis especially among pregnant women.

## Methods

### Study site

The Brong Ahafo region is one of the ten regions of Ghana. It is the second largest region and covers an area of 39,557 sqkm. Health delivery in the region is currently centred on 27 functional administrative districts with an estimated population of 2,418,511 and an annual population growth rate of 2.3% [8].

The region shares common boundaries with five others–Northern region to the north, Ashanti and Western regions to the south, Volta region to the east and to the Eastern region to the South East. It also shares an international boundary to the west with La Côte d’Ivoire [9].

**Figure 1:**
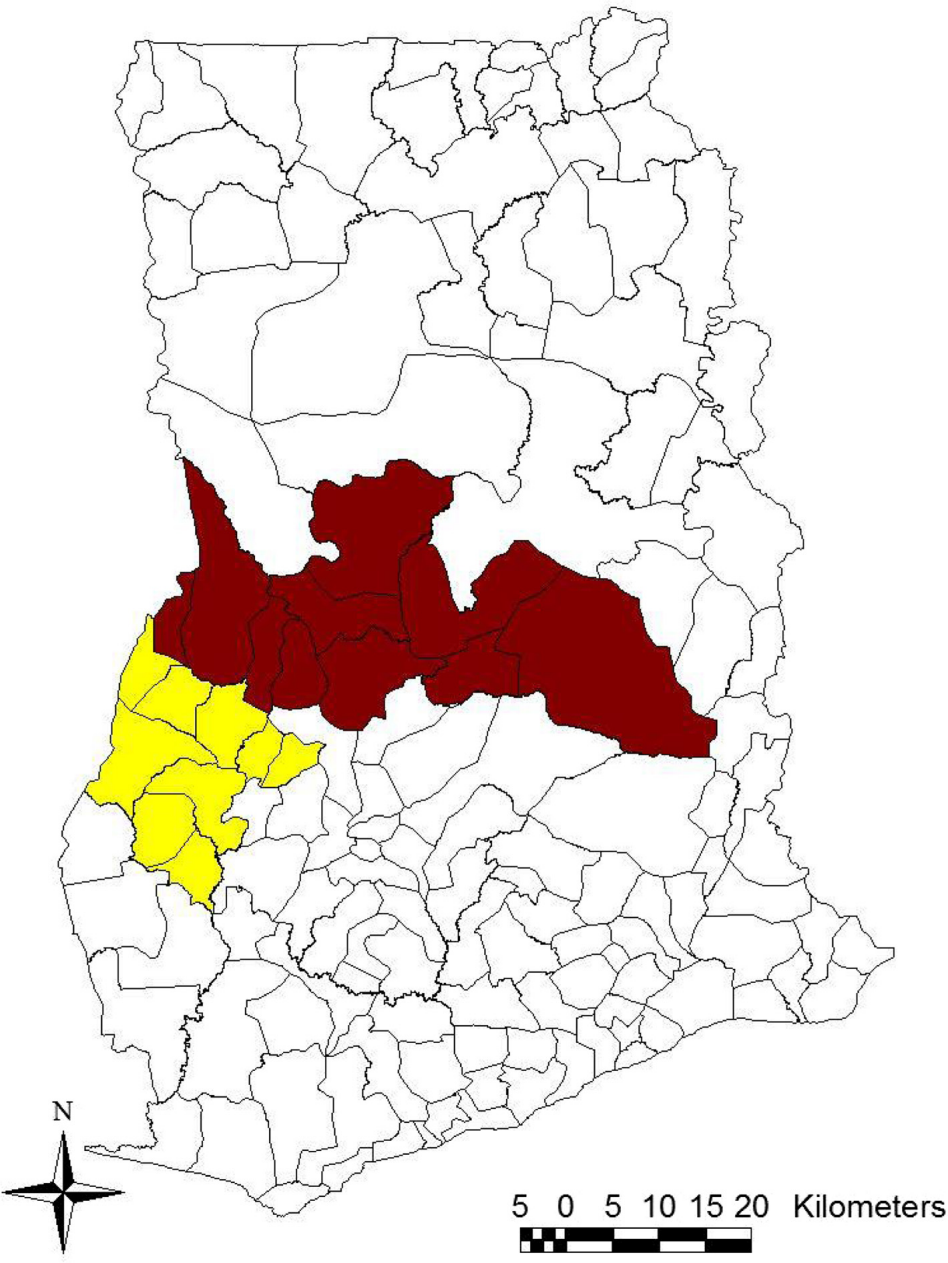
**A map of Ghana with Brong Ahafo region showing districts in the two ecological zones: red indicates districts in the savanna transition zone and yellow showing districts in the forest zone.**

The districts in the region were increased from 22 to 27 in 2012 but the new districts at the time of the study did not have fully functional administrative structures. For that matter, the old demarcation was used for the purpose of this study. There are two main ecological zones in the region, namely the forest and savanna transition zones with 11 districts in each ecological zone (Figure 1).

The total number of antenatal registrants in the region in 2012 was 97,384 [Brong Ahafo regional health directorate, 2012, unpublished observation]. Though as high as 82,776 (85%) were tested for HIV only 41,378 (41%) of these women were screened for syphilis; this was slightly higher than the national average of 40.3% [NACP annual report, 2012, unpublished]. Within the region, the savanna transition zone, however, recorded lower syphilis screening uptake than the forest zone.

### Study design

A cross-sectional study was conducted in two districts within the savannah transition zone, identified as having recorded the highest and the lowest syphilis screening to assess health facility and individual level factors that affect syphilis screening among pregnant women.

### Sample size and sampling procedure

Two districts (**Kintampo North and Techiman)** in the west transition zone were selected. Kintampo municipality recorded the highest syphilis screening (39.2%) while Techiman municipality had the lowest screening (25.8%) in this zone based on 2012 district level data [2012 annual report, Brong Ahafo region, unpublished observation]. All health facilities providing ANC services in the two districts were included to assess their level of preparedness to carry out syphilis screening. Record review was conducted in each of the health facilities on number of ANC registrants in 2012, number screened for syphilis, evidence of ongoing screening and evidence of availability of syphilis test kits.

For the individual level factors, the sample size was 390 pregnant women. This sample was proportionately allocated depending on the total number of pregnant women registered at each hospital ANC in 2012.

Over 70% of pregnant women attend antenatal services at the hospitals in Brong Ahafo [Brong Ahafo region 2012 report, unpublished]. For the community level factors therefore, pregnant women attending ANCs run by hospitals within the selected districts formed the target population.

The sample size was distributed proportionately among the hospitals. Participants who consented to participate in the study were selected after the general health education prior to the start of individual level consultation for the day through random sampling. The sample for each day was selected through balloting. The total ballots were indicated as selected or not selected based on the total number of women present during the general health education and the sample required for the day till the required number was achieved. Data collectors conducted “Exit interviews” for those who were selected.

One standardized questionnaire was used to collect data from health facilities in the two districts. The questionnaire captured information related to syphilis screening on each health facility. An interview was also conducted with the health personnel who directly attend to antenatal women in the participating facilities and contained questions on their training and management of syphilis. Review of records of pregnant women registry was done retrospectively for 2013.

The second tool was a questionnaire designed to identify client level factors that influence syphilis screening. It collected information on socio-demographic characteristics of expectant mothers, obstetric history, their knowledge and attitude toward syphilis screening. Knowledge about syphilis was measured by the following questions “How does one get syphilis?” “Does syphilis affect the unborn baby?” “Can syphilis be treated?” “Should pregnant women be screened for syphilis?” each of the responses was assigned “0” for no answer “-1” for wrong answer and “+1” for correct answer. The scores were summed and “positive” was given if the total score was > 0 and “negative” if it was <0 (Modified version from Munkhuu et al., [10]. Trained field workers from Kintampo Health Research Centre administered the questionnaire in a language in which the respondents could communicate. In instances where a respondent could not communicate in any of the dominant languages, a local translator who spoke English was used.

### 3.9 Statistical analysis

For the health system factors, the primary outcome was syphilis screening status of health facilities at the time of the survey. All health facilities that provided ANC in 2013 and had complete data on syphilis screening were considered in the analysis. Prevalence of syphilis screening was defined as fraction of pregnant women who were screened for syphilis. The independent factors considered in this part were type of ownership, district, and level of health care, availability of resources and personnel as well as health education concerning syphilis at the clinic. Pearson chi-squared test was used to examine the relation between syphilis screening status and each of the factors. Where cell frequencies were few Fisher’s exact test was used.

The standard questionnaire administered to the 390 pregnant women measured individual level factors associated with syphilis screening. In this data, the outcome of interest was syphilis screening status of pregnant women defined as having screening result, which was seen and confirmed by the interviewer. The exposure variables considered were socio-demographic, obstetric and health seeking characteristics of pregnant women. All pregnant women who had complete data on syphilis screening were included in the analysis. Logistic regression was used to investigate factors that were associated with syphilis screening. First, the association between syphilis screening and each of the potential factors were examined using a univariate logistic regression model. All variables from the univariate logistic regression model that were found to be statistically significant (p < 0.05) were included in a multivariate logistic regression model. Likelihood ratio p-values were reported for categorical variables with more than two exposure levels while Wald p-values were reported for binary variables. The analyses were performed using STATA Version 11 (Statcorp, College station, Texas, USA).

Approval for this study was obtained from ethics committee of the Ghana health service and written permission was obtained from the two district directors of the participating districts.

## Results

In all 37 health facilities conducted antenanal clinics in the two districts in 2013, 75.7% of the health facilities were public; Techiman had the higher number of health facilities (64.9%), test kits were available in only 29.7% of the health facilities and 43.2% of 37 health facilities were conducting syphilis screening Community health planning and services (CHPS) facilities were in the majority (37.8%) followed by health centers (28.7%) while hospitals were the least (13.5%). Over 75% (28) of these health facilities were publicly owned and at the time of the survey, 70% of the 37 facilities did not have syphilis screening test kits, 78% without appropriate drugs for treatment of syphilis and 86% did not have guidelines for managing syphilis in pregnancy. Of the total health facilities in the two districts, 51.3% had qualified personnel for syphilis screening, 45.9% had their staff trained on syphilis screening and only 35.1% had more than one trained staff for syphilis. However, ANC based syphilis screening was being offered in only 43.2% of the health facilities surveyed. Ten (10) out of the 16 health facilities that were offering syphilis screening frequently had shortage of test kits and only 1 health facility never ran out of screening materials.

### Syphilis testing

Table 1 shows Proportion of pregnant women screened for syphilis in different types of health facilities in Brong Ahafo Region in 2013. In all, 18,292 pregnant women were registered across the two districts in 2013 in the record review. Approximately, 67% of the women were registered in Techiman municipality. About 45% of the women were registered by private health care providers. Hospitals registered the highest number (42%) followed by maternity homes (30%) while Community Health Planning and services (CHPS) zones recorded the least (10%). About 52% of 18,292 pregnant women were screened for syphilis. Of these, 102 (1.1%) tested serologically positive. This represents syphilis prevalence of 1.06% in the two districts. The two municipalities recorded approximately similar proportions of women screened for syphilis; about 48% for Kintampo and 52% for Techiman respectively. Public health facilities recorded 82.7% of screening compared to 15.9% by private health facilities. Though 30% of the pregnant women were registered at the maternity homes, only 2.6% were screened for syphilis while CHPS zones did not screen pregnant women.

**Table 1:** **Proportion of pregnant women screened for syphilis in different types of health facilities in Brong Ahafo region 2013**

**Type of health facility**	**Number of pregnant women registered (%)**	**Number of women screened for syphilis (%)**
**District**		
Kintampo	5176 (28.30)	2482 (47.95)
Techiman	13116 (71.70)	7100 (54.13)
**Ownership**		
Public	9992 (54.62)	8264 (82.71)
Private	8300 (45.38)	1318 (15.88)
**Level of health facility**		
Hospital	7718 (42.19)	7659 (99.24)
Health center	3039 (16.61)	1178 (38.76)
Maternity home	5580 (30.50)	145 (2.60)
CHIPS	1955 (10.69)	-
**Total**	18292 (100)	9582 (52.38)

### Health facility level factors affecting screening of pregnant women for syphilis

Table 2 displays the distribution of health facilities and syphilis screening status by background characteristics. Screening of women was more likely to occur in hospitals and (80%, p=0.003), in health facilities with test kits (81%, p=0.002), in health facilities with qualified personnel (78.9%, p < 0.001), where ANC clinics were run by doctors (100%, p < 0.001), in health facilities that organized syphilis training for their staff (76.5%, p < 0.001), in health facilities that had more than one trained staff (92.3%, p < 0.001) and where syphilis education was being offered at the ANC level (53.8%, p=0.045) (Table 2).

**Table 2:** **Distribution of health facilities and syphilis screening status by background characteristics**

**Characteristic**	**Number of health facilities (N=37)**	**Number of facilities screening for syphilis n (%)**	**Number of health facilities not screening (%)**	**Chi-square p-value**
**Name of district**				p=0.068
Kintampo	13	3 (23.1)	10 (76.9)	
Techiman	24	13 (54.2)	11 (45.8)	
**Type of health facility**				*p=0.003*
CHPS	14	1 (7.4)	13 (92.6)	
Maternity home	7	3 (42.9)	4 (58.1)	
Health center	11	8 (72.3)	3 (27.3)	
Hospital	5	4 (80.0)	1 (20.0)	
**Type of Hospital ownership**				p=0.391
Public	28	11 (39.3)	17 (60.3)	
Private	9	5 (55.6)	4 (44.4)	
**Availability of test kits**				*p=0.002*
No	26	7 (26.9)	19 (73.1)	
Yes	11	9 (81.8)	2 (18.2)	
**Availability of drugs**				p=0.711
No	29	13 (44.8))	16 (55.2)	
Yes	8	3 (37.5)	5 (62.5)	
**Availability of guidelines/protocols**				p=0.416
No	32	13 (40.6)	19 (59.4)	
Yes	5	3 (60.0)	2 (40.0)	
**Availability of qualified personnel for screening**				*p < 0.001*
No	18	1 (5.6)	17 (94.4)	
Yes	19	15 (78.9)	4 (21.1)	
**Type of health care personnel in charge**				*p=0.003*
Community health nurse	16	2 (12.5)	14 (87.5)	
Midwife	20	13 (65.0)	7 (35.0)	
Doctor	1	1 (100)	0 (00.0)	
**Training on syphilis screening**				*p < 0.001*
No	20	3 (15.0)	17 (85.0)	
Yes	17	13 (76.5)	5 (23.5)	
**Number of staff trained on screening**				*p < 0.001*
0	20	3 (15.0)	17 (85.0)	
1	4	1 (25.0)	3 (75.0)	
2 or more	13	12 (92.3)	1 (7.7)	
**Syphilis education at ANC**				
No	11	2 (18.2)	9 (81.8)	*p=0.045*
Yes	26	14 (53.8)	12 (46.2)	

Bivariate associations determined by chi-square tests at 5% Significance level.

### Individual level factors

In this part of the study 390 pregnant women participated and all were included in the analysis. The mean age of the participants was 26.9 (SD=2.5) years. In general, 345 (89.4%) women were older than 19 years, 291 (74.8%) were of Christian religion, while 302 (77.4%) were married, 247 (63.3%) had studied beyond junior high school level and 131 (33.1%) of them were engaging in trading activities. Most (207,53.8%) of the participants were in their third trimester of pregnancy. Over half of the participants (262,67.7%) had a previous pregnancy before, but only 106 (27.2%) had attended ANC in the previous pregnancy with 116 (29.7%) having some knowledge about syphilis. Majority of the respondents attended public hospital (64.4%). Of the total participants, 306 (78.6%) never received syphilis education in the course of their pregnancies, while only 78 (20.0%) received syphilis education during the index visit and 157 (40.5%) were willing to request for syphilis screening if they were not offered at the clinic. However the proportion of syphilis screening among the participants was only 23.1% (95% CI; 19.0, 27.6).

### Individual and community level factors influencing screening of pregnant women for syphilis

Table 3 describes socio-demographic and antenatal care factors associated with screening among antenatal women attending hospitals in Brong Ahafo region. The results indicated that pregnant women who were screened were more likely to be in their third trimester as compared with those in the first trimester (31.1%, p < 0.001) and would either have completed senior high education and above (34.0%, p=0.001) or be in the trading business (33.6%, p=.001). Also the screened group were significantly more likely to live in Techiman municipality (28.3%, p < 0.001), attending antenatal clinics run by public hospitals (31.3%, p < 0.001), particularly Holy Family hospital (50.8%, p < 0.001), and had received syphilis education during antenatal visits (38.1%, p < 0.001), especially the index visit (34.6%, p=0.007) and were generally more likely to request for syphilis screening (31.1%, p=0.001).

**Table 3:** **Socio-demographic and antenatal care factors associated with screening among antenatal women attending hospitals in Brong Ahafo region**

**Characteristic**	**Total (N=290)**	**Not syphilis screened n (%)**	**Syphilis screened n (%)**	**P-value**
**Age group (years)**				0.516
15-19	41	34 (82.9)	7 (17.1)	
20-29	225	173 (76.9)	52 (23.1)	
30-49	120	89 (74.2)	31 (23.3)	
**Religion**				0.599
Christianity	291	221 (76.0)	70 (24.1)	
Islam	96	76 (79.2)	20 (20.8)	
Traditional	2	2 (100.0)	0 (00.0)	
**Marital statu**s				
Single	85	65 (76.5)	20 (23.5)	0.634
Married	302	232 (96.8)	70 (23.2)	
Divorced/separated	3	3 (100)	0 (00.0)	
**Educational status**				0.001
Primary/none	86	73 (84.9)	13 (15.1)	
JHS	56	48 (85.7)	8 (14.3)	
SHS	153	101 (66.0)	52 (34.0)	
Tertiary	94	77 (81.9)	17 (18.1)	
**Employment**				0.001
Housewife/unemployed	88	68 (77.3)	20 (22.7)	
Farmer	51	45 (88.2)	6 (11.8)	
Trader	131	87 (66.4)	44 (33.6)	
Artisan	82	64 (78.1)	18 (21.9)	
Professional	38	36 (94.7)	2 (5.26)	
**Gestational age**				*<0.001*
1st trimester	51	50 (98.0)	1 (2.0)	
2nd trimester	127	105 (82.7)	22 (17.3)	
Third trimester	207	142 (68.60)	65 (31.4)	
**Parity**				0.252
None	125	96 (76.8)	29 (23.2)	
1-3	218	163 (74.8)	55 (25.2)	
4+	44	44 (86.4)	6 (13.6)	
**ANC in a previous pregnancy**				0.693
Yes	284	217 (76.41)	67 (23.6)	
No	106	83 (78.3)	23 (21.7)	
**know syphilis and ANC screening**				0.467
Yes	116	208 (75.9)	66 (24.1)	
No	274	92 (79.3)	24 (20.7)	
**District**				*<0.001*
Kintampo	125	110 (88.0)	15 (12.0)	
Techiman	265	190 (76.9)	75 (28.3)	
**Received ANC at:**				*<0.001*
Private hospital	139	128 (92.1)	11 (7.9)	
Public hospital	251	172 (68.5)	79 (31.5)	
**Which hospital do you attend ANC**				*<0.001*
Mount Olive	29	28 (96.6)	1 (3.4)	
Ahamadiya	53	49 (92.5)	7 (7.5)	
OpokuAgyemang	57	51 (89.5)	6 (10.5)	
Kintampo	125	110 (88.0)	15 (12.0)	
Holy family	126	62 (49.2)	64 (50.8)	
**Ever Received syphilis education in this pregnancy?**				*<0.001*
No	306	248 (81.1)	58 (18.9)	
Yes	84	52 (61.9)	32 (38.1)	
**Syphilis talk during this visit?**				*0.007*
No	311	248 (79.7)	63 (20.3)	
Yes	78	51 (63.4)	27 (34.6)	
**Will you request for syphilis screening?**				*0.001*
No	231	191 (82.7)	40 (17.3)	
Yes	157	108 (68.8)	49 (31.2)	
**Total screened**			89 (23.1, 95% CI=19.0-27.6)	

Bivariate associations determined by chi-square tests at 5% Significance level.

Table 4 displays significant factors associated with syphilis screening among pregnant women. The study also showed that women who attended public hospitals were 5 times more likely to be screened than those who attended private hospitals (OR=5.49; 95% CI 1.71-17.65). Also, women who were willing to request for syphilis screening had higher chances of getting screened than those who were not (OR=2.72; 95% CI 1.26-5.88) and women in their third trimester are more likely to be screened than those in their first trimester (OR=9.09; 95% CI 1.09-75.58) (Table 4).

**Table 4:** **Significant factors associated with syphilis screening among pregnant women from final multiple logistic regression model**

**Characteristics**	**Adjusted odds ratio (AOR) (95% CI)**	**p-value**
**Type of ownership**		0.004**
Private	Ref	
Public	5.49 (1.71-17.65)	
**Pregnant women willing to request for screening**		
No	Ref	0.011**
Yes	2.72 (1.26-5.88)	
**Gestational age**		
1st trimester	Ref	0.0015*
2nd trimester	9.09 (1.09-75.58)	
3rd trimester	16.47 (2.02-132.81)	

*Likelihood ratio test **Wald test.

## Discussion

Though antenatal based screening for syphilis is free and being offered at the point of care in the country, close to half of all the pregnant women who reported at these 37 health facilities did not get the service. All but one of the facilities that offered the screening reported frequent shortage of test kit. This resulted in almost half of the women visiting ANCs in the study area without receiving syphilis screening. One in every 100 women tested serologically positive and 97% of them were treated, but information on contact tracing was not available.

The 52% coverage reported in this study is higher than previously reported for the country (40%) [11] and other parts of sub-Saharan Africa (38%) [12], but lower than coverage in India (57%) [13] and Mongolia (77%) [10]. The national AIDS/STI control program plans to screen 80% of all pregnant women reporting at medical facilities in the country by 2015. These targets could be achieved in urban areas because women in those parts of the country are served by hospitals where screening coverage is high. However, achieving similar results in rural communities could prove difficult because such communities are served more by CHPS compounds and health centers where syphilis screening capacity is limited [13].

The 1.1% prevalence reported in this study is much lower than previously reported in the country and other parts of Africa [14-16]. However this result could have underestimated the true prevalence because most women who were screened were more likely to be urban dwellers where the prevalence is reported to be lower than women in rural areas as a result of limited access to medical treatment among rural dwellers [17]. Notwithstanding this low prevalence, routine ANC base screening is still recommended because its effectiveness has been established even in low prevalence settings [18].

Screening for syphilis was limited in lower level health facilities compared with hospitals. The Ghana’s health sector reforms established three levels of care at the district/municipality level. The CHPS concept which was created in 2005 as a means of extending coverage of basic primary health care to all citizens serve mostly rural communities and managed by community health officers who carryout public health interventions with limited resources and training. Clinics/health centers serve larger communities with trained physician assistants and midwives while hospitals serve as primary referral centers with better resources for comprehensive and uninterrupted health care as compared with CHPS and Clinics [13]. However, the introduction of point of care testing, which requires no laboratory capacity, could improve uptake of syphilis screening even in remote rural communities by building the capacity of community health workers [19].

Syphilis screening status of health facilities did not differ significantly between public and private health facilities. However, while 80% of women got screened in public health facilities, close to 75% of the women who obtained their services at private health facilities were not screened even though test kits were being provided free of charge to all facilities, implying low screening by private health practitioners despite service availability. Many other logistics such as gloves, syringes and needles are required for the screening but are not provided by the program and this would suggest additional resources have to be committed by the various institutions for screening without cost recovery. While this might pose little problem for public health institutions that are under state subvention, private health providers will have low motivation to commit their resources into a screening program without direct benefit, thus accounting for low uptake among private health providers. This has a serious implication for the target set my NACP, especially in districts where private health facilities dominate. To ensure full participation of the private sector in scaling up ANC based health programs the policy should be repackaged to incorporate incentives that target private health practitioners.

Availability of test kits and trained, qualified personnel were found to be associated with screening and health facilities with more than one trained personnel more likely to screen in this study. This is necessary to ensure uninterrupted screening. A Tanzanian study identified insufficient supply of test kits, lack of trained personnel, frequent transfer of trained personnel before replacement or no cover for leave and sickness as reasons for low uptake of ANC base screening [20]. Therefore successful implementation of syphilis screening programmes can be achieved through the primary health care system (PHC) if such programmes are given the necessary impetus through training, supervision and resources [12].

Also, ANC clinics run by doctors and midwives were more likely to screen for syphilis than those run by community health nurses. Poor health workers’ understanding behind screening, a common phenomenon among middle and lower level health workers may influence their decision to screen pregnant women [12]. However, the observation in this study could possibly be due to differential availability of resources at the health facilities as doctors and midwives are likely to work in hospitals with better logistics.

### Community/Individual level factors

In this part of the study, the proportion of pregnant women screened for syphilis was markedly lower than that of the health facilities data. The data relates to women who had been screened at the time of the interview (May 2014) and may reflect the rate of screening at that time. Though this study was conducted exclusively among pregnant women who used hospitals as their ANC and therefore had higher chances of being screened, less than 25% of them had been screened which was far lower than reported elsewhere [10,12,13]. The disparity between this figure and the proportion of screening in respect to health facility level data probably reflects the erratic nature of logistic supply for the program in Ghana. For example, at the time of the data collection, most of the hospitals that reported screening in the first part of this study did not have test kits and women who reported being screened might have had screening done in the early part of the year when test kits were available. This supports the findings that the odds of screening was higher among women in their third trimester of pregnancy, contrary to Munkhuu et al., [10], who identified third trimester as a risk factor for not being screened. It is a serious concern that women who visit health facilities in their first and second trimesters of pregnancy are not being screened for syphilis. Identification and treatment of syphilis is only effective if it is performed early in pregnancy [21]. Therefore our findings indicate a missed opportunity to avert the adverse outcomes of syphilis in pregnancy.

Willingness to request for syphilis screening was a significant individual level factor for a woman to be screened. Because the study was an exit interview this finding could be due to post-test counseling of those who had the screening done; thereby giving them better understanding of the effects of syphilis.

Interestingly, knowledge about syphilis did not affect screening in this study. Previous studies identified poor knowledge about syphilis and its effects in pregnancy as potential barriers to syphilis screening [13,22]. But these studies were conducted at a time when the screening was outside the ANC and participants had to pay for the test. Under such settings knowledge of complications of the disease will motivate women to screen. In the current context, women are routinely offered screening services when it is available at the point of care without cost. Evidence in Ghana suggests that women do not opt out of free services that are offered at health facilities, because of growing confidence in the health worker [23].

Level of education did not significantly influence screening among pregnant women. Munkhuu et al., [10], reported similar observations in Mongolia. This is contrary to another finding that, women with higher level of education were more likely to utilize maternal health services in a rural state of Nigeria than those with lower educational status [24]. Education is critical to women gaining knowledge about the effects of syphilis to their health and health of their babies, which could influence their desire to request for screening. However, the point of care screening in Ghana is health worker-driven and they provide education on syphilis, thus level of formal education probably does not play a significant role in this setting.

### Limitations of the study

Our findings should be interpreted with caution in the light of several limitations. Firstly, the study was conducted in only two districts with few number of health facilities. The sample size was too small to allow in-depth analysis of potential factors associated with syphilis screening, especially at the health facility level. Larger sample size would have given this study enough statistical power to perform sub-group analysis to determine the relationship between syphilis screening and potential confounders. Nonetheless, being the first of its kind in Ghana and Africa, for that matter, the findings will serve as a baseline for future studies. Secondly, limiting the survey to only women attending ANCs run by hospitals may also affect generalizing the result to the entire districts since most women attending hospitals are likely to live around the cities and may have different socio-demographic characteristics from women living in rural settings. Thirdly, timing of syphilis screening for pregnant women, which is critical in prevention of congenital syphilis, was not determined [25]. This occurred as a result of participants’ poor recall of time of screening. Early screening in pregnancy and treatment of positive women is effective in syphilis prevention programmes [26]. Finally, because of the structured nature of the study, limited potential factors were evaluated. A qualitative method would have produced potential factors which were probably left unevaluated but that would have had implication for generalizability.

## Conclusion and recommendations

This study provides evidence of the existence of large gaps between ANC coverage and syphilis screening in Brong Ahafo region, despite the policy to freely screen pregnant women. These disparities exist because the lower level health facilities lack human resource and logistical capacity, creating a missed opportunity to screen pregnant women who use those services. Lack of motivation has also been suggested to hamper full private sector participation in the implementation of the screening programme. The findings particularly highlight insufficient supply of test kits and lack of training, especially for peripheral health workers as hindrances to universal coverage of ANC base screening.

We therefore recommend training of health workforce, especially at the periphery and empowering them with the essential logistics as critical for rapid scale-up of syphilis screening. Regular supply of logistics is crucial to the success of the programme. Therefore, regular procurement and timely distribution of tests kits is highly recommended.

The interest of private health care providers should be incorporated into the programme by providing them with free accessory screening materials such as gloves, needles and syringes.

### Consent

Written informed consent was obtained from the patient’s guardian/parent/next of kin for the publication of this report and any accompanying images.

## Abbreviations

ANC: Antenatal care/clinic
CHPS: Community base health planning and services
HIV: Human immunodeficiency virus
MTCT: Mother-to-child transmission
NACP/STI: National Aids/Sexually Transmitted Infections Programme
PMTCT: Prevention of mother-to-child transmission
RDHS: Regional directorate of health services
WHO: World Health Organization
